# TGF-β-induced IL-6 prevents development of acute lung injury in influenza A virus-infected F508del CFTR-heterozygous mice

**DOI:** 10.1152/ajplung.00078.2015

**Published:** 2015-04-03

**Authors:** Parker S. Woods, Mia F. Tazi, Nicholas M. Chesarino, Amal O. Amer, Ian C. Davis

**Affiliations:** ^1^Department of Veterinary Biosciences, The Ohio State University, Columbus, Ohio;; ^2^Department of Microbial Infection and Immunity, The Ohio State University, Columbus, Ohio

**Keywords:** acute lung injury, cystic fibrosis transmembrane conductance regulator, interleukin-6, influenza, transforming growth factor-β

## Abstract

As the eighth leading cause of annual mortality in the USA, influenza A viruses are a major public health concern. In 20% of patients, severe influenza progresses to acute lung injury (ALI). However, pathophysiological mechanisms underlying ALI development are poorly defined. We reported that, unlike wild-type (WT) C57BL/6 controls, influenza A virus-infected mice that are heterozygous for the F508del mutation in the cystic fibrosis transmembrane conductance regulator (HETs) did not develop ALI. This effect was associated with higher IL-6 and alveolar macrophages (AMs) at 6 days postinfection (d.p.i.) in HET bronchoalveolar lavage fluid (BALF). In the present study, we found that HET AMs were an important source of IL-6 at 6 d.p.i. Infection also induced TGF-β production by HET but not WT mice at 2 d.p.i. TGF-β neutralization at 2 d.p.i. (TGF-N) significantly reduced BALF IL-6 in HETs at 6 d.p.i. Neither TGF-N nor IL-6 neutralization at 4 d.p.i. (IL-6-N) altered postinfection weight loss or viral replication in either mouse strain. However, both treatments increased influenza A virus-induced hypoxemia, pulmonary edema, and lung dysfunction in HETs to WT levels at 6 d.p.i. TGF-N and IL-6-N did not affect BALF AM and neutrophil numbers but attenuated the CXCL-1/keratinocyte chemokine response in both strains and reduced IFN-γ production in WT mice. Finally, bone marrow transfer experiments showed that HET stromal and myeloid cells are both required for protection from ALI in HETs. These findings indicate that TGF-β-dependent production of IL-6 by AMs later in infection prevents ALI development in influenza A virus-infected HET mice.

influenza a viruses cause a highly contagious acute respiratory disease in humans, which is the eighth leading cause of attributable annual mortality in the USA. It has been estimated that seasonal influenza infections result in 12 influenza-related deaths per 100,000 persons per year in the United States, most of which occur in the elderly (22, 48). The emergence of a novel H1N1 swine flu influenza A virus strain in 2009 resulted in a worldwide pandemic and caused an estimated 14,800 excess deaths in the United States alone (9). Emergent highly pathogenic H5N1 and H7N9 avian influenza A strains pose a continuing danger to the human population, particularly as they have very high mortality rates (>60%) (45, 60).

Vaccines and neuraminidase inhibitors are routinely used for influenza prophylaxis and treatment, respectively (11). However, there are significant limitations to vaccine development, uptake, and efficacy (41, 42, 50). Indeed, poor matching between the 2014 influenza vaccine strains and circulating seasonal influenza A viruses may have contributed to the high severity of the 2014–2015 influenza season (23, 51). Antiviral drugs such as oseltamivir may reduce influenza severity and transmission early in infection (32). However, development of viral resistance mutants is rapid, and these drugs are increasingly viewed as poorly effective (12, 18).

Approximately 20% of patients with severe influenza develop acute lung injury (ALI), which is associated with poor prognosis (35). Once ALI has developed, the only treatment option is nonspecific supportive management in an intensive care unit. This is often of limited efficacy, and death from ALI in patients with severe influenza is common (49, 67). New drugs are needed to treat late-stage influenza (2). However, development of such drugs requires a better understanding of basic mechanisms underlying the pathogenesis of influenza-induced ALI (53).

Alveolar type II epithelial cells are the main infection target and site of replication for influenza viruses in the distal lung (30, 61). A primary physiological function of these cells is to regulate the depth of the thin (8 to 10 μm) layer of fluid lining the bronchoalveolar space. This process is dependent on the relative magnitude of active Na^+^ absorption via epithelial Na^+^ channels (ENaC) and Cl^−^ absorption or secretion through the cystic fibrosis transmembrane conductance regulator (CFTR) anion channel (15). Compromised ion transport leads to pulmonary edema and hypoxemia and is associated with poor outcomes in patients with ALI (66).

We reported previously that increased CFTR-mediated anion secretion played a role in development of pulmonary edema in influenza A virus-infected mice (68). We have shown that, unlike in wild-type (WT) C57BL/6 controls, influenza A virus infection did not cause ALI in C57BL/6-congenic mice that are heterozygous for the F508del mutation (a phenylalanine deletion at position 508) in CFTR (HETs) (1). This effect was associated with higher bronchoalveolar lavage fluid (BALF) IL-6 content and alveolar macrophage (AM) counts at 6 days postinfection (d.p.i.) in HETs. Notably, AM depletion in HETs increased ALI severity to WT levels at 6 d.p.i. and concomitantly reduced BALF IL-6 levels by almost 80%.

IL-6 can play both pro- and anti-inflammatory roles in ALI pathogenesis (17, 34, 44, 69). Given the strong correlation between increased BALF IL-6 and absence of ALI in influenza-infected HETs, the aim of this study was to define the source of this cytokine and its contribution to protection from ALI in HETs. By performing cytokine neutralization and bone marrow transfer experiments, we found that TGF-β produced early in response to influenza infection prevents ALI development in HET mice by inducing AM production of IL-6 later in infection. These results indicate that alterations in CFTR expression and/or function significantly impact the innate immune response of AMs to influenza viruses and further emphasize the role of CFTR as an important modulator of the host immune response (1, 2, 68).

## MATERIALS AND METHODS

### 

#### Breeding and genotyping of F508del mice.

HETs and WT controls were generated by breeding B6.129S7-Cftr^*tm1Kth*^ mice (70). All procedures were approved by the Institutional Animal Care and Use Committee at The Ohio State University. Ethical considerations precluded performance of survival studies.

#### Infection of mice.

WT and HET mice (8–12 wk old) were intranasally infected with 10,000 plaque-forming units (pfu)/mouse of egg-grown influenza A/WSN/33 (H1N1) in 50 μl PBS with 0.1% BSA (2, 63). In our hands, this inoculum induces ALI in WT mice by 2 d.p.i. and results in 100% mortality by 8 d.p.i. (median time to death: 7 days) (2, 68), without replication in the brain (3). At the time of infection, mice were individually marked and then weighed every other day. Data for each experimental group were derived from a minimum of two independent infections.

#### Immunohistochemistry.

Thin sections (3 μm) were prepared from formalin-fixed, paraffin-embedded lung tissue (31). IL-6 was detected using a goat polyclonal antibody (AB-406-NA; R & D Systems, Minneapolis, MN). Bound antibody was detected using biotinylated anti-goat immunoglobulin (Vector Laboratories, Burlingame, CA), the Vectastain ABC peroxidase system, and 3,3′-diaminobenzidine substrate (Vector Laboratories). Sections were counterstained with Harris hematoxylin, scanned with a Scanscope CS slide scanner (Aperio Technologies, Vista, CA), and visualized with ImageScope software (Aperio Technologies).

#### Quantitative real-time PCR.

BALF AMs were isolated by adherence to polystyrene as in our prior studies (25). Isolated AMs were lysed, and total RNA was extracted using the RNeasy system (Qiagen, Alameda, CA). RNA was reverse transcribed into cDNA using the high-capacity cDNA reverse transcription kit (Applied Biosystems, Grand Island, NY). Quantitative real-time PCR amplification of cDNA was performed using the TaqMan Gene Expression system (Applied Biosystems). Expression of *il-6* mRNA was determined by the ΔΔCt method and normalized to the endogenous control *18s rrna*, which was not altered by infection (26).

#### Antibody-mediated cytokine neutralization.

To neutralize TGF-β, mice were treated with a single dose of a polyclonal TGF-β-neutralizing antibody (AB-100-NA; 100 μg/mouse in 100 μl saline; R & D Systems) at 2 d.p.i. by intraperitoneal (i.p.) injection (7). To neutralize IL-6, mice were treated i.p. with a single dose of a polyclonal antibody to IL-6 (AB-406-NA; 0.5 μg/mouse in 100 μl saline; R & D Systems) at 4 d.p.i. (56). Controls were treated i.p. with rabbit IgG at both time points (100 μg/mouse in 100 μl saline; Southern Biotech, Birmingham, AL).

#### Bone marrow transfer.

As in our previous studies, bone marrow-recipient mice were irradiated with 1,000 cGy given in two doses by a ^137^Cs irradiator (3). Twenty-four hours later, donor mice were euthanized, and hind limb long bones were flushed with conditioned media to isolate low-density bone marrow. Fresh bone marrow cells (5 × 10^6^/mouse) (in 0.5 ml PBS) were then transplanted by tail vein injection into recipient mice, which were placed on Baytril antibiotic water bottles for 2–3 wk peri-irradiation. This protocol resulted in no outward clinical signs.

#### Additional methods.

Bronchoalveolar lavage and measurements of carotid arterial O_2_ saturation, heart rate, lung homogenate viral titers, lung wet:dry weight ratios, lung mechanics, and BALF inflammatory mediators were performed as in our previous studies (14, 68).

#### Statistical analysis.

Descriptive statistics were calculated using Instat 3.05 (GraphPad Software, San Diego, CA). Gaussian data distribution was verified by the method of Kolmogorov and Smirnov. Differences between group means were analyzed by one-way ANOVA, with Tukey-Kramer multiple-comparison posttests. *P* < 0.05 was considered statistically significant. All data are presented as means ± SE.

## RESULTS

### 

#### HET AMs produce more IL-6 than WT AMs in response to influenza A virus infection.

**Fig. 1.**
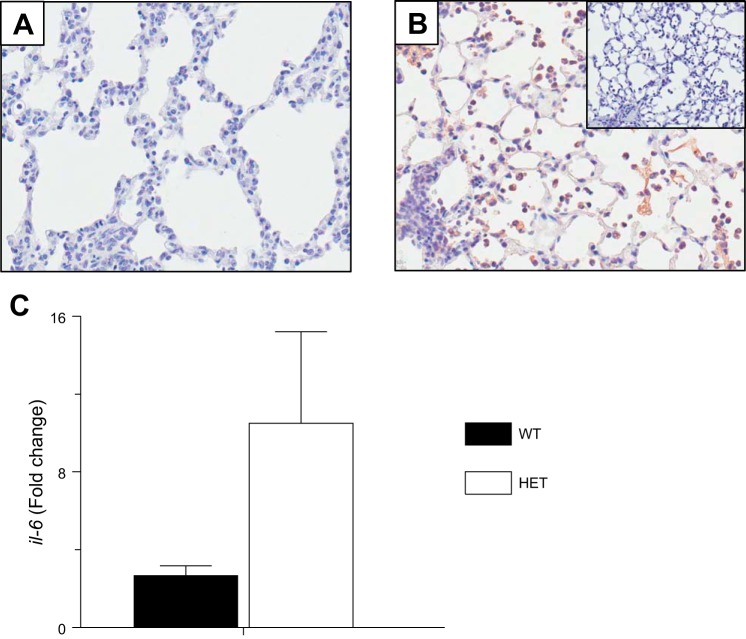
Alveolar macrophages (AMs) from influenza A virus-infected mice that are heterozygous for the F508del mutation in the cystic fibrosis transmembrane conductance regulator (HET) produce more IL-6 than wild-type (WT) AMs in response to influenza A virus infection. *A*: representative immunohistochemistry for IL-6 at 6 days postinfection (d.p.i.) in formalin-fixed, paraffin-embedded lung tissue from a WT mouse intranasally infected with influenza A/WSN/33 [10,000 plaque-forming units (pfu)/mouse; original objective lens magnification ×40]. *B*: representative immunohistochemistry for IL-6 in formalin-fixed, paraffin-embedded lung tissue from a HET mouse at 6 d.p.i. (original objective lens magnification ×40; *inset*: tissue stained with nonspecific polyclonal goat antibody). *C*: fold change in *il-6* gene expression in AMs isolated from WT mice (*n* = 11) and HET mice (*n* = 13) at 6 d.p.i., relative to uninfected WT mice (*n* = 4) and HET mice (*n* = 3). *P* < 0.005, vs. WT mice. Data are presented as means ± SE.

We reported previously that attenuation of cardiopulmonary dysfunction in HETs infected with the A/WSN/33 (H1N1) influenza strain (10,000 pfu/mouse) was associated with exaggerated AM and IL-6 responses at 6 d.p.i. (1). We also showed that HET BALF IL-6 was reduced by ∼80% following depletion of AMs using clodronate liposomes, indicating that the exaggerated IL-6 response to influenza A virus in HETs was AM dependent. We therefore wished to determine the extent to which IL-6 was AM derived in infected HETs. IL-6 was not detected in lung tissue sections from WT mice at 6 d.p.i. by immunohistochemistry (Fig. 1*A*). In contrast, the majority of AMs in HET lung tissues were IL-6 positive at 6 d.p.i. (Fig. 1*B*). No antigen-positive AMs were detected in tissues immunostained with a nonspecific control antibody. Lastly, quantitative real-time PCR demonstrated that, relative to uninfected controls of the same strain, influenza A virus infection induced significantly higher *il-6* gene expression in AMs isolated from HET mice than AMs from WT mice at 6 d.p.i. (Fig. 1*C*).

#### The exaggerated IL-6 response of HETs to influenza A virus infection is TGF-β dependent.

**Fig. 2.**
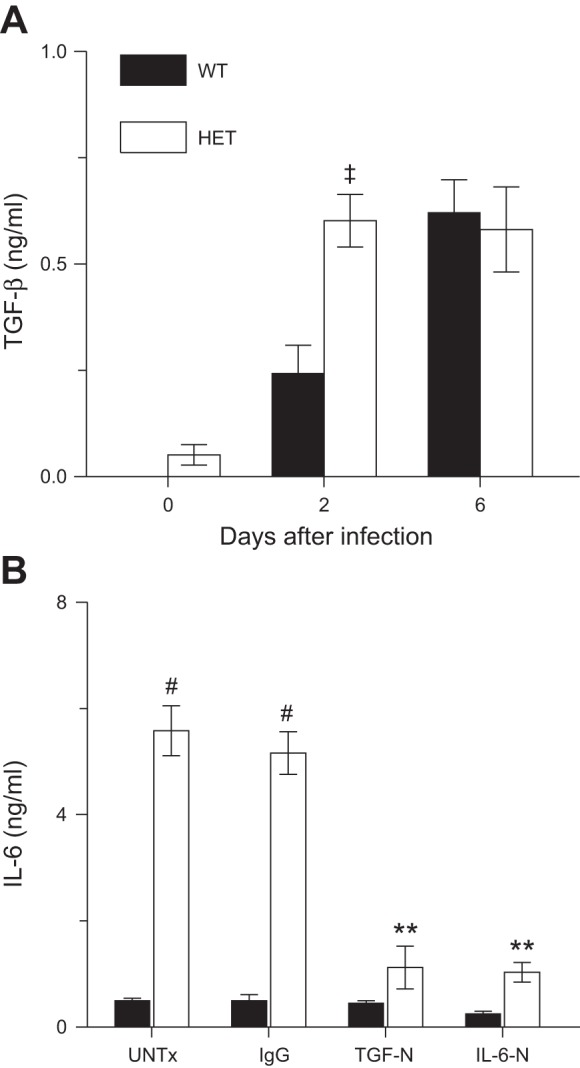
The exaggerated IL-6 response of HETs to influenza infection is TGF-β dependent. *A*: effect of influenza A/WSN/33 infection on bronchoalveolar lavage fluid (BALF) TGF-β in WT controls (*n* = 10–14) and HETs (*n* = 10–12). *B*: effect of intraperitoneal treatment with nonspecific IgG (IgG; 100 μg/mouse), treatment with a neutralizing antibody to TGF-β at 2 d.p.i. (TGF-N; 100 μg/mouse), or treatment with a neutralizing antibody to IL-6 at 4 d.p.i. (IL-6-N; 0.5 μg/mouse) on BALF IL-6 in WT mice (*n* = 8) and HETs (*n* = 8) at 6 d.p.i. UNTx: untreated animals. ‡*P* < 0.005, vs. WT mice at the same time point. **P < 0.005, #*P* < 0.001, vs. WT mice in the same treatment group. Data are presented as means ± SE.

Given the known role of TGF-β as an anti-inflammatory cytokine (55) and an inducer of IL-6 (4, 21), we extended these observations by determining effects of the HET genotype on the TGF-β response to influenza A virus infection. We found that BALF TGF-β levels were higher in influenza A virus-infected HETs at 2 d.p.i. (Fig. 2*A*). BALF TGF-β increased further in WT but not HET mice at 6 d.p.i., resulting in no difference between mouse strains at this time point.

To determine whether the exaggerated IL-6 response to infection in HET mice was TGF-β dependent, influenza A virus-infected mice were treated with a single dose of a neutralizing antibody to TGF-β at 2 d.p.i. (TGF-N) (7). Control groups were untreated or treated with nonspecific IgG. TGF-N had no significant effect on BALF TGF-β at 6 d.p.i. in either WT or HET mice (not shown) but reduced BALF IL-6 by ∼80% in HETs at this time point (Fig. 2*B*). This was comparable to that caused by AM depletion in HETs at 6 d.p.i. (1). Treatment with a single dose of a neutralizing antibody to IL-6 at 4 d.p.i. (IL-6-N) reduced BALF IL-6 at 6 d.p.i. in both mouse strains although we cannot exclude the possibility that this effect was a result of competition between the neutralizing and ELISA antibodies to IL-6.

#### Neutralization of TGF-β or IL-6 increases severity of cardiopulmonary dysfunction in influenza A virus-infected HETs without significantly impacting viral replication.

**Fig. 3.**
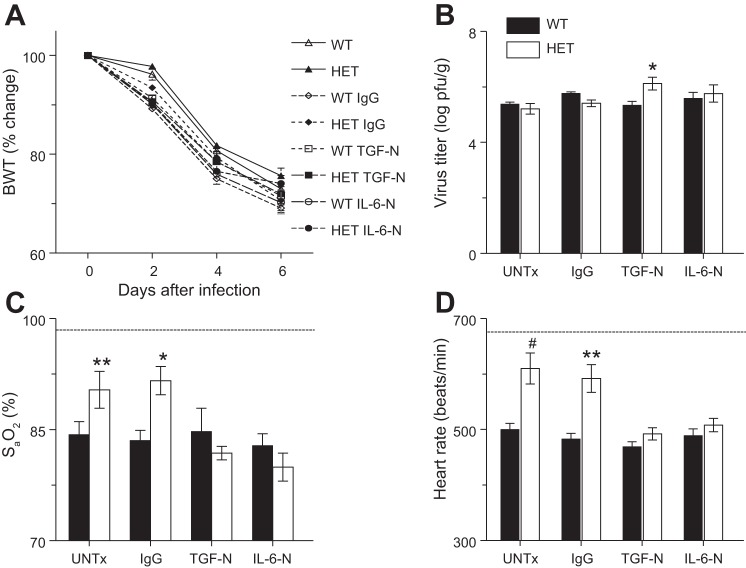
Neutralization of TGF-β or IL-6 increases severity of cardiopulmonary dysfunction in influenza-infected HETs without significantly impacting viral replication. Effects of systemic treatment with nonspecific IgG, TGF-N, and IL-6-N on body weight (BWT; % change from *day 0*; *n* > 10 per group) (*A*), viral titers in lung homogenates at 6 d.p.i. (log pfu/g; *n* = 5–8 per group) (*B*), carotid arterial oxygen saturation (S_a_O_2_) at 6 d.p.i. (*n* > 10 per group) (*C*), and heart rate at 6 d.p.i. (*n* > 10 per group) (*D*). Dashed line indicates mean value for each parameter in uninfected WT mice. *P < 0.05, **P < 0.005, #*P* < 0.001, vs. WT mice in the same treatment group. Data are presented as means ± SE.

To determine whether altered TGF-β and IL-6 responses to infection contribute to protection from influenza A virus-induced ALI, we examined the effects of TGF-N and IL-6-N on weight loss, viral replication, and cardiopulmonary function in influenza A/WSN/33 virus-infected mice at 6 d.p.i. We selected this time point because it would allow us to directly compare the downstream effects of treatment with anti-TGF-β at 2 d.p.i. with those of treatment with anti-IL-6 at 4 d.p.i. Postinfection weight loss at 6 d.p.i. did not differ between untreated WT and HET mice (Fig. 3*A*). IgG treatment, TGF-N, and IL-6-N modestly accelerated weight loss in both strains. However, these effects were not statistically significant. Likewise, viral replication was not significantly affected by IgG treatment or IL-6-N in either strain (Fig. 3*B*). Viral titers in HET mice were significantly higher than in WT controls following TGF-N although the difference in titers was <1 log and thus is unlikely to be of biological significance. Importantly, both TGF-N and IL-6-N increased the severity of influenza A/WSN/33 virus-induced hypoxemia (Fig. 3*C*) and bradycardia (Fig. 3*D*) in HET mice but not WT controls at 6 d.p.i. IgG treatment had no effect on carotid arterial oxygen saturation or heart rate in either strain at this time point.

#### Neutralization of TGF-β or IL-6 in HETs increases severity of influenza A virus-induced pulmonary edema to WT levels.

**Fig. 4.**
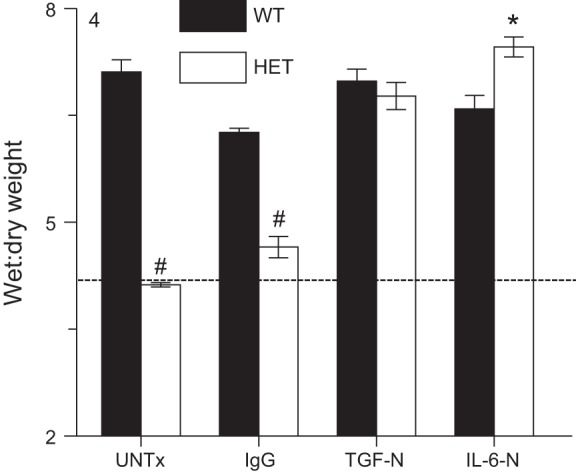
Neutralization of TGF-β or IL-6 in HETs increases severity of influenza A virus-induced pulmonary edema to WT levels. Effects of systemic treatment with nonspecific IgG, TGF-N, and IL-6-N on lung water content (wet:dry weight ratio) at 6 d.p.i. Dashed line indicates mean value for each parameter in uninfected WT mice; *n* ≥ 8 per group. *P < 0.05, #*P* < 0.001, vs. WT mice in the same treatment group. Data are presented as means ± SE.

In the absence of treatment, lung water content (wet:dry weight) remained normal (comparable to uninfected values) in HET mice at 6 d.p.i. but was significantly elevated in WT controls (Fig. 4). Both TGF-N and IL-6-N significantly increased lung water content at 6 d.p.i. in HETs but not WT controls, resulting in equally severe pulmonary edema in both strains at this time point. IgG had no such effect.

#### TGF-β and IL-6 are necessary to maintain normal lung function in influenza-infected HETs.

**Fig. 5.**
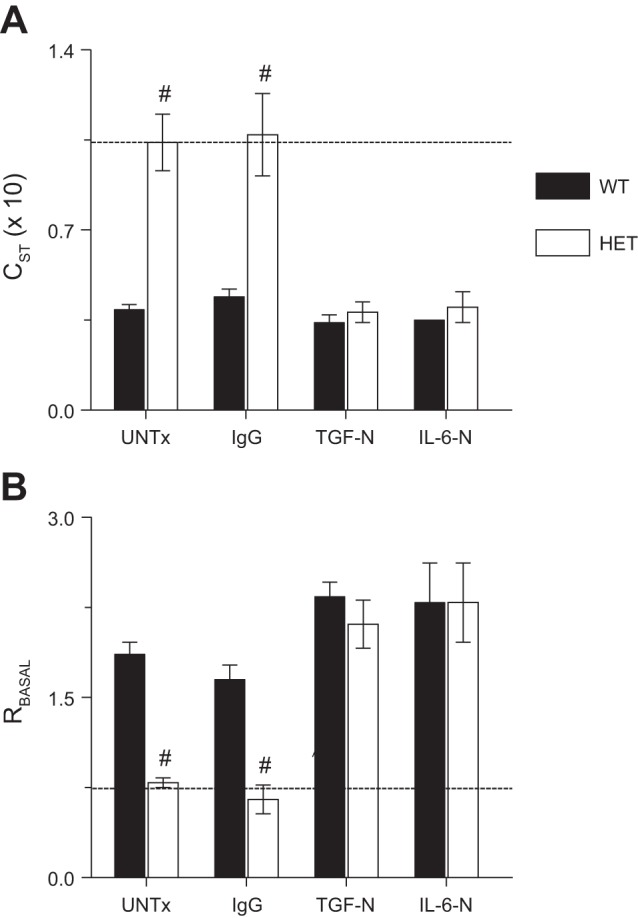
TGF-β and IL-6 are necessary to maintain normal lung function in influenza-infected HETs. Effects of systemic treatment with nonspecific IgG, TGF-N, and IL-6-N on static lung compliance (C_ST_; ml/cmH_2_O, × 10) at 6 d.p.i. (*A*) and baseline total lung resistance (R_BASAL_; cmH_2_O·s^−1^·ml^−1^) at 6 d.p.i. (*B*). Dashed line indicates mean value for each parameter in uninfected WT mice. *n* = 6–8 per group. #*P* < 0.001, vs. WT mice in the same treatment group. Data are presented as means ± SE.

In the absence of treatment or after treatment with IgG, static and dynamic lung compliance were both significantly higher (and essentially normal) in infected HETs at 6 d.p.i. (Fig. 5*A* and data not shown, respectively). Likewise, total lung resistance was not higher in untreated or IgG-treated HETs than uninfected WT mice at 6 d.p.i. but was greatly increased in influenza A virus-infected WT controls at this time point (Fig. 5*B*). Protective effects of the HET genotype on static lung compliance, dynamic lung compliance, and airway resistance were completely abrogated by TGF-N and IL-6-N, which resulted in comparable values to WT mice.

#### Neutralization of TGF-β or IL-6 does not impact leukocyte infiltration of the lung in response to influenza infection.

**Fig. 6.**
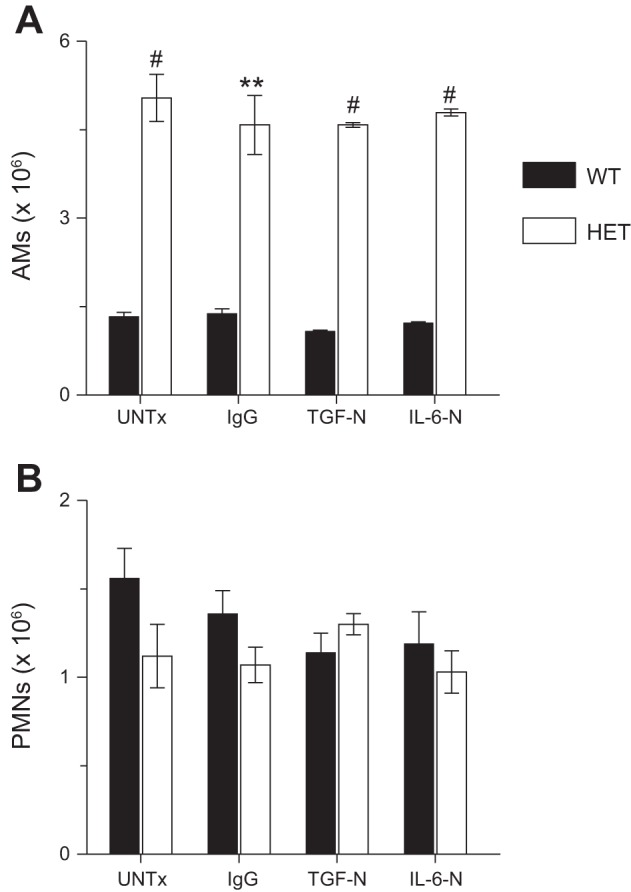
Neutralization of TGF-β or IL-6 does not impact leukocyte infiltration of the lung in response to influenza infection. Effects of systemic treatment with nonspecific IgG, TGF-N, and IL-6-N on BALF AMs (× 10^6^) at 6 d.p.i. (*A*) and BALF neutrophil counts (polymorphonuclear leukocytes, PMNs; × 10^6^) at 6 d.p.i. (*B*); *n* = 5–7 per group. **P < 0.005, #*P* < 0.001, vs. WT mice in the same treatment group. Data are presented as means ± SE.

As in our earlier studies, BALF from influenza A/WSN/33 virus-infected HET mice contained far greater numbers of AMs than WT controls at 6 d.p.i. (Fig. 6*A*). However, BALF neutrophil counts did not differ between strains at this time point (Fig. 6*B*). Interestingly, IgG treatment, TGF-N, and IL-6-N had no effect on BALF AM and neutrophil numbers in either mouse strain at 6 d.p.i.

#### Neutralization of TGF-β and IL-6 alters bronchoalveolar lavage fluid chemokine and cytokine responses to influenza infection.

We previously reported that BALF IFN-γ, IL-10, CC chemokine ligand 2 (CCL-2)/monocyte chemotactic protein 1 (MCP-1), CCL-5/RANTES, and CXCL-10/inducible protein 10 content did not differ between WT and HET mice at 6 d.p.i. (1). However, HET BALF contained larger amounts of the neutrophil chemoattractant CXCL-1/keratinocyte chemokine (KC). In the present study, we also found similar levels of IFN-γ, IL-10, CCL-2/MCP-1, and CCL-5/RANTES in untreated infected animals from both strains at 6 d.p.i. (Table 1 and data not shown). However, CXCL-1/KC levels did not differ between untreated HETs and WT controls in these experiments. Interestingly, we also found that BALF IL-12 content (which we had not previously measured) was almost fourfold higher in untreated HETs at 6 d.p.i.

**Table 1.** Neutralization of TGF-β or IL-6 modulates chemokine and cytokine responses to influenza A virus infection at 6 d.p.i.

Genotype	Treatment	CXCL-1	IFN-γ	IL-12	CCL-5
WT	None	210 ± 10	1450 ± 250	280 ± 40	120 ± 10
	IgG	220 ± 40	1320 ± 180	240 ± 40	120 ± 20
	TGF-N	**50 ± 20**^c^	**320 ± 80**^c^	**2030 ± 380**^c^	**40 ± 10**^c^
	IL-6-N	**50 ± 10**^c^	**130 ± 30**^c^	**2100 ± 650**^b^	140 ± 20
HET	None	170 ± 10	2370 ± 220^d^	1070 ± 160^e^	150 ± 30
	IgG	170 ± 10	2660 ± 340^d^	1360 ± 200^f^	150 ± 10
	TGF-N	**40 ± 10**^c^	1820 ± 190	1660 ± 200	160 ± 20^f^
	IL-6-N	**70 ± 10**^c^	2350 ± 460	**1940 ± 190**^a^	130 ± 10

Values are means ± SE, pg/ml (to the nearest significant figure), *n* = 4–6 per group. IgG treatment was with nonspecific rabbit IgG. Antibody-mediated neutralization of TGF-β was at 2 days postinfection (d.p.i.). Antibody-mediated neutralization of IL-6 was at 4 days d.p.i.

WT, wild-type C57BL/6 mice; HET, C57BL/6-congenic mice heterozygous for the F508del cystic fibrosis transmembrane conductance regulator mutation; N, neutralization.

a *P* < 0.05,

b *P* < 0.005,

c *P* < 0.001, vs. untreated mice of the same genotype (boldface font).

d *P* < 0.05,

e *P* < 0.005,

f *P* < 0.001, vs. WT mice in the same treatment group.

TGF-N and IL-6-N did not alter BALF IL-10 and CCL-2/MCP-1 levels at 6 d.p.i. in either WT mice or HETs (not shown). However, both treatments significantly reduced BALF CXCL-1/KC content in both mouse strains. In WT mice, both TGF-N and IL-6-N also significantly reduced BALF IFN-γ but increased IL-12 at 6 d.p.i. TGF-N, but not IL-6-N, also reduced BALF CCL-5/RANTES in WT mice at this time point. In contrast, neither treatment impacted BALF IFN-γ or CCL-5/RANTES in HET mice, and only IL-6-N significantly increased BALF IL-12 at 6 d.p.i. Treatment with nonspecific IgG had no effect on CXCL-1/KC, IFN-γ, IL-12, or CCL-5/RANTES in either mouse strain.

#### Both stromal and myeloid cells from HET mice are necessary for protection from ALI and exaggerated secretion of IL-6.

**Fig. 7.**
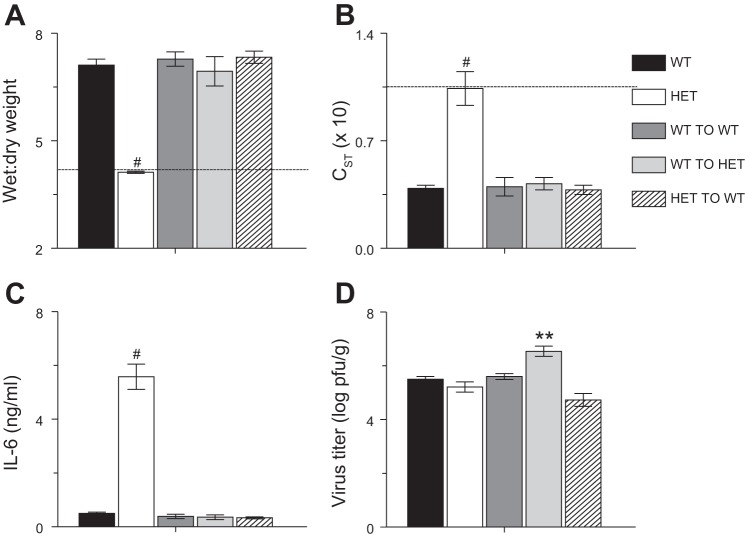
Both stromal and myeloid cells from HET mice are necessary for protection from acute lung injury and exaggerated secretion of IL-6. Effects of reciprocal bone marrow transfer on lung water content (wet:dry weight ratio) at 6 d.p.i. (*A*), static lung compliance (C_ST_; ml/cmH_2_O, × 10) at 6 d.p.i. (*B*), BALF IL-6 (ng/ml) at 6 d.p.i. (*C*), and viral titers in lung homogenates at 6 d.p.i. (log pfu/g) (*D*). WT TO WT: transfer of WT donor bone marrow to WT recipient mice. WT TO HET: transfer of WT donor bone marrow to HET recipient mice. HET TO WT: transfer of HET donor bone marrow to WT recipient mice. Dashed line indicates mean value for each parameter in uninfected WT mice; *n* = 5–7 per group. **P < 0.005, #*P* < 0.001, vs. WT mice. Data are presented as means ± SE.

We previously demonstrated that HET AMs were necessary for protection from influenza A virus-induced ALI in HET mice (1). To determine the extent to which interactions between AMs and respiratory epithelial cells were necessary for induction of IL-6 production at high levels by HET AMs at 6 d.p.i., we performed reciprocal bone marrow transfers between WT and HET mice. We found that, relative to WT controls, transfer of WT bone marrow to WT mice had no effect on lung water content (Fig. 7*A*), static lung compliance (Fig. 7*B*), dynamic lung compliance (not shown), and total lung resistance (not shown). WT-to-WT bone marrow transfer also had no effect on BALF IL-6 content (Fig. 7*C*) or viral replication (Fig. 7*D*). These data indicate that the irradiation and bone marrow transfer procedures had no effect on the response of WT mice to influenza A virus infection. In contrast, both transfer of WT bone marrow to HET mice and transfer of HET bone marrow to WT mice increased lung water content to WT levels. This effect was accompanied by a reduction in BALF IL-6 to WT levels. Viral replication did not differ between groups, with the exception of HET recipients of WT bone marrow, whose lung homogenate viral titers were ∼1 log higher than WT controls. Because of limited availability of HET mice, HET-to-HET control bone marrow transfer experiments were not performed.

## DISCUSSION

Despite a 50% reduction in cell-surface CFTR expression and anion transport (20), we and others have reported that uninfected HETs have normal lung function and exhibit no immune abnormalities (1, 13). However, we have demonstrated that, unlike WT controls, influenza A virus-infected HET mice did not develop ALI despite comparable viral replication kinetics and weight loss in both strains (1). This protective phenotype was associated with exaggerated AM and IL-6 responses at 6 d.p.i. In the present study, we extended these observations by investigating the role of IL-6 in protection from ALI in infected HET mice. We found that AMs were a major source of IL-6 in HETs at 6 d.p.i. Infection also induced TGF-β secretion by HET but not WT mice at 2 d.p.i. Importantly, treatment with a single dose of a TGF-β-neutralizing antibody at 2 d.p.i. significantly reduced BALF IL-6 in HETs at 6 d.p.i. This indicated that IL-6 production by HET AMs at this time point was TGF-β dependent. Neither TGF-N nor treatment with a single dose of a neutralizing antibody against IL-6 at 4 d.p.i. significantly altered the rate of postinfection weight loss or the magnitude and kinetics of viral replication in either mouse strain. In contrast, both treatments increased the severity of hypoxemia, pulmonary edema, and lung dysfunction in HETs to WT levels. TGF-N and IL-6-N did not affect BALF AM and neutrophil numbers in either strain. However, both treatments attenuated the CXCL-1/KC response (which is important for neutrophil recruitment to the lung) in both mouse strains. TGF-N and IL-6-N also reduced IFN-γ production in WT mice. Finally, bone marrow transfer experiments showed that HET stromal and myeloid cells are both required for protection from ALI in HETs. Together, our data show that TGF-β-dependent production of IL-6 by AMs later in influenza infection prevents ALI development in HETs.

Previous investigators have reported that an attenuated AM response contributes to increased influenza severity (29, 59, 64). We showed previously that AMs were required for protection from ALI in infected HETs, which supports these earlier findings (1). We also showed that AM depletion reduced BALF IL-6 to WT levels at 6 d.p.i., which suggested that IL-6 production was AM dependent in HETs. However, because IL-6 can be produced by multiple cells, these data did not definitively indicate whether macrophages were the actual source of IL-6 or simply necessary for induction of IL-6 production by other cells in the distal lung. Likewise, we could not determine whether AMs, IL-6, or both were necessary to prevent development of ALI in HETs. In the present study, we found that TGF-N and IL-6-N did not change BALF AM and neutrophil levels in HETs at 6 d.p.i., yet both treatments reduced BALF IL-6 and increased the severity of ALI to WT levels at this time point. Importantly, TGF-N and IL-6-N did not alter ALI severity in WT mice at 6 d.p.i., which indicates that the effects of cytokine neutralization were specific. Taken together, these data indicate that, although increased AM recruitment to the lungs of HET mice is not TGF-β or IL-6 dependent, AMs are necessary for protection from development of ALI in HETs because they release large amounts of IL-6 at 6 d.p.i. in response to TGF-β produced at 2 d.p.i. However, because bone marrow transfer experiments show a role for stromal cells in protection from ALI in HETs, we cannot exclude an additional role for alveolar epithelial cells in IL-6 production.

We found no differences in viral replication kinetics and BALF neutrophil counts between WT mice, untreated or IgG-treated HETs, and antibody-treated HETs at 6 d.p.i. This indicates that, in our model, influenza-induced ALI is independent of these factors. Moreover, we did not find that development of ALI in HETs following TGF-N and IL-6-N was associated with development of a so-called “cytokine storm” (10, 16, 33, 62). For instance, both TGF-N and IL-6-N decreased BALF IFN-γ in WT mice, yet neither treatment had any effect on ALI severity in this mouse strain. In contrast, both TGF-N and IL-6-N induced ALI in HET mice without altering BALF IFN-γ content.

Some investigators have reported an association between high BALF IL-6 and increased influenza mortality (37, 39, 56). However, others have found that disease severity is not reduced in IL-6-knockout mice (43, 54, 58) or that IL-6 is protective (6, 17, 65). Our data support this latter conclusion, as do our earlier studies, which showed a correlation between increased BALF IL-6 at 6 d.p.i. and amelioration of ALI following treatment of influenza-infected WT mice with the de novo pyrimidine synthesis inhibitor A77-1726 (2). Hence, we find that increased IL-6 production is associated with protection from ALI in both genetic and therapeutic models. However, the specific cell type producing IL-6 was not defined in our earlier experiments. AMs have been shown to produce IL-6 in response to in vitro infection with highly pathogenic influenza viruses (46). Likewise, AM production of IL-6 has been inferred from clodronate liposome-mediated AM depletion experiments (1, 39) although the underlying mechanisms by which AM production of IL-6 was induced were not investigated.

Greater influenza severity has also been linked to inadequate anti-inflammatory responses. TGF-β is a potent inflammatory regulator (55), and increased lung levels of active TGF-β have been linked to reduced influenza morbidity and mortality (7, 8, 19, 40). We found previously that attenuation of influenza A virus-induced ALI in WT mice treated with A77-1726 was temporally associated with elevated BALF TGF-β (2). Importantly, TGF-β has been shown to induce IL-6 (4, 21), which is consistent with our data. Unfortunately, however, because both TGF-N and IL-6-N resulted in comparable effects on the infected HET lung, we were unable to determine whether TGF-β can also contribute to ALI attenuation in influenza-infected HETs in an IL-6-independent fashion. Moreover, we have yet to define the cellular mechanism(s) by which a 50% reduction in CFTR expression and function in HETs leads to earlier TGF-β production after influenza infection although studies in patients with CF suggest that reduced CFTR expression and/or function results in increased TGF-β production (24).

We have yet to determine the pathophysiological processes that underlie protection from ALI by TGF-β and/or IL-6 in influenza. Alveolar type II respiratory epithelial cells are the main infection target and site of replication for influenza viruses in the distal lung (30, 61). In addition to synthesizing surfactant lipids and proteins, a primary function of these cells is to regulate the depth of the bronchoalveolar lining fluid. ENaC and CFTR channels expressed on the apical surface of bronchoalveolar epithelial cells play a central role in this process (5, 15, 38). Importantly, TGF-β can downregulate epithelial CFTR expression and function (27, 28, 47, 52, 57). Moreover, IL-6 can stimulate ENaC activity (36). We have previously shown that influenza infection results in activation of A_1_-subtype adenosine receptors, which induces increased CFTR-mediated Cl^−^ secretion (68). Hence, we propose that ALI does not develop in influenza-infected HETs as a result of three factors. First, the capacity for CFTR-mediated Cl^−^ secretion by HET respiratory epithelial cells in response to A_1_-adenosine receptor activation is inherently reduced as a result of a 50% decrease in CFTR expression (20). Second, higher TGF-β production early in infection will further inhibit CFTR-mediated Cl^−^ secretion by HET respiratory epithelial cells and also induces IL-6 production by HET AMs. Third, higher levels of IL-6 in the HET lung at 6 d.p.i. will result in greater stimulation of ENaC-mediated Na^+^ absorption compared with WT mice. Together, these effects will result in reduced alveolar edema and improved lung function. However, we cannot yet exclude the possibility that IL-6 acts upstream of some other protective mediator.

In conclusion, our findings indicate that IL-6 secreted by AMs in response to epithelial TGF-β prevents development of ALI in influenza-infected HETs. By extension, our data imply that inadequate AM, TGF-β, and/or IL-6 responses may contribute to the development of ALI following infection by highly pathogenic influenza strains. These results indicate that CFTR and IL-6 are both important host determinants of influenza severity and suggest that CFTR may have potential as a target for development of novel treatments for influenza-induced ALI.

## GRANTS

P. Woods was supported by The Ohio State University Howard Hughes Medical Institute Med-to-Grad Training Program and the C. Glenn Barber Fund. M. Tazi was supported by a Careers in Immunology Fellowship from the American Association of Immunologists. N. Chesarino was supported by The Ohio State University Systems and Integrative Biology Training Program Grant from the National Institute of General Medical Sciences at the National Institutes of Health (T32-GM068412). A. Amer was supported by a Cystic Fibrosis Foundation Research Grant and The Ohio State University Public Health Preparedness in Infectious Diseases Program. I. Davis was supported by The Ohio State University Public Health Preparedness in Infectious Diseases Program and The National Heart Lung and Blood Institute at the National Institutes of Health (R01-HL102469).

## DISCLOSURES

No conflicts of interest, financial or otherwise, are declared by the authors.

## AUTHOR CONTRIBUTIONS

Author contributions: P.S.W., M.F.T., and N.M.C. performed experiments; P.S.W., M.F.T., N.M.C., and I.C.D. analyzed data; P.S.W. and I.C.D. interpreted results of experiments; P.S.W. and I.C.D. prepared figures; A.O.A. and I.C.D. edited and revised manuscript; A.O.A. and I.C.D. approved final version of manuscript; I.C.D. conception and design of research; I.C.D. drafted manuscript.
